# Stabilization of Loess Using Nano-SiO_2_

**DOI:** 10.3390/ma11061014

**Published:** 2018-06-14

**Authors:** Ran Kong, Fanyu Zhang, Gonghui Wang, Jianbing Peng

**Affiliations:** 1 MOE Key Laboratory of Mechanics on Disaster and Environment in Western China, Department of Geological Engineering, Lanzhou University, Lanzhou 730000, China; Kongran413@163.com; 2 Research Center on Landslides, Disaster Prevention Research Institute, Kyoto University, Gokasho, Uji, Kyoto 611-0011, Japan; wanggh@landslide.dpri.kyoto-u.ac.jp; 3 Department of Geological Engineering, Chang’an University, Xi’an 710054, China

**Keywords:** loess, nano-SiO_2_, strength enhancement, structure modification

## Abstract

Improving the performance of loess is of significant importance for lowering its collapsibility and water sensitivity to construction requirements and for geohazard mitigation. The present paper studies the changes in mechanical, structural, and mineralogical properties of nano-SiO_2_-treated loess with different contents and curing days. The mechanical behavior was examined by unconfined compressive strength (UCS) of untreated and treated loess. To better understand the mechanisms of stabilization, particle size distributions, scanning electron microscope (SEM) images, and X-ray diffraction (XRD) analyses were carried out. The results show that the UCS increase with increasing contents and curing days due to nano-SiO_2_ addition produced coarser particles, denser packing, and smaller pores in treated loess. The changes in the properties can be attributed to the formation of aggregation and agglomeration, with greater particle sizes and more interparticle contact. In addition, the results from mineralogical component analysis further confirm that physical structure modification controls the changes in mechanical and fabric properties, rather than chemical component alteration. Even small nano-SiO_2_ additions can also provide great improvement when curing days are enough for the treated loess. These findings reveal that nano-SiO_2_ has the potential to serve as a cost-effective stabilized additive that treats the universal loess.

## 1. Introduction

Loess is a widespread surface deposit in many parts of the world. It is an important engineering material, for example as the material in a filled embankment. It is a typically problematic soil due to its propensity to collapse and subsidence after loading and wetting. It is also a typically hazardous material due to landslide and erosion prevalence. Therefore, it has a need for improvement by various stabilization methods, such as construction requirements and geohazard mitigation.

There has been considerable research into using various stabilizing agents to improve the performance of loess but much of this effort has focused on traditional chemical additives. Lime, fly ash, and cement are commonly used materials to reach more durable loess in infrastructure construction and geological barriers [1,2,3,4,5,6]. Overall, these chemical additives cause short-term physiochemical modification with a decrease in water content and an increase in density. The chemical reactivity then causes long-term pozzolanic stabilization with mineralogical and structural changes [2,6]. As a result, these chemical additives produce stronger and more applicative loess in mechanics and physics terms. However, they also produce some chemical changes in treated loess [6]. Typically, these chemical additives cause much greater alkaline and saline environments, due to an increase in pH and salinity [1,4,6]. In the soil improvement field, greater and more urgent attention has been paid to carbon emissions and treatment costs regarding various industries which involve lime, cement, and fly ash [7,8]. Hence, there has been great interest in replacing the generally-used chemical additives in soil improvement with alternative materials.

The interest in alternative materials has increased greatly in soil improvement works [9,10]. Nano-particles have also received special attention due to their unique properties [11,12]. Previous research has shown that nano-particles result in changes to physical, mechanical, and chemical properties. Taha and Taha [13] investigated the effect of nano-particles on clay properties. They found that nano-clay, nano-alumina, and nano-copper additives strikingly restrain the expansive and shrinkage behaviors of clays and do not change its mineralogical properties. Research on nano-copper oxide and gamma-aluminum oxide powder on clay properties showed lower conductivity and higher shrinkage due to smaller pores and more flocculated fabric [14,15]. The addition of the nano-particles can reduce the development of desiccation cracks in clays [13,14]. At the same time, the addition of nano-clay causes a decrease in Atterberg limits of clays [13,16,17]. Furthermore, a small amount of nano-SiO_2_ addition can produce an obvious increase in the strength of treated clay [17]. The aforementioned research work has focused on clays, even though silty and sandy soils are also often used in civil engineering. For these soils, increasing interest in the modification of their various properties following nano-particles addition has been conducted by several researchers. Gallagher and Mitchell showed that colloidal silica clearly decreases the risk of liquefaction of loose sand under seismic loading [18]. Huang and Wang [19] studied the impact of laponite on the strength of silty sand to mitigate liquefaction occurrence. Ren and Hu [20] investigated the effect of nano-SiO_2_ on silty soil properties. Tabarsa, Latifi [21] evaluated the feasibility of loess improvement using nano-clay based on laboratory and field investigations. On the whole, the addition of nano-particles results in higher strength and density and lower conductivity, shrinkage, and Atterberg limit in various treated soils.

Nano-particles have been investigated as alternative additives for soil improvement purposes in replacing traditional stabilization agents. To some extent, their high cost limits their widespread application. However, nano-particles may generate lower total cost in practical engineering than traditional chemical additives, such as concrete and lime [20]. Therefore, nano-particles have more advantages and greater potential to become cost-effective additives. In addition, the potential advantages in nano-particles would be a promising additive in soil improvement. Nevertheless, the use of nano-particles as a soil stabilizer is still uncommon [15]. Until now, there have been very few attempts at using a nano-particle additive to treat loess [21].

The present paper shows results from the addition of nano-SiO_2_ into loess by examining changes in mechanical, mineralogical, and structural properties with different nano-SiO_2_ contents and curing days. A series of tests were conducted on unconfined compressive strength, particle size distributions, SEM images, and XRD analyses. This study aims to understand the improvement of nano-SiO_2_ treated loess along with the complicated relations between macroscopic behaviors and microscopic characteristics. It is important for the improvement mechanisms and practical applications of treated loess.

## 2. Materials and Methods

### 2.1. Tested Materials

**Figure 1:**
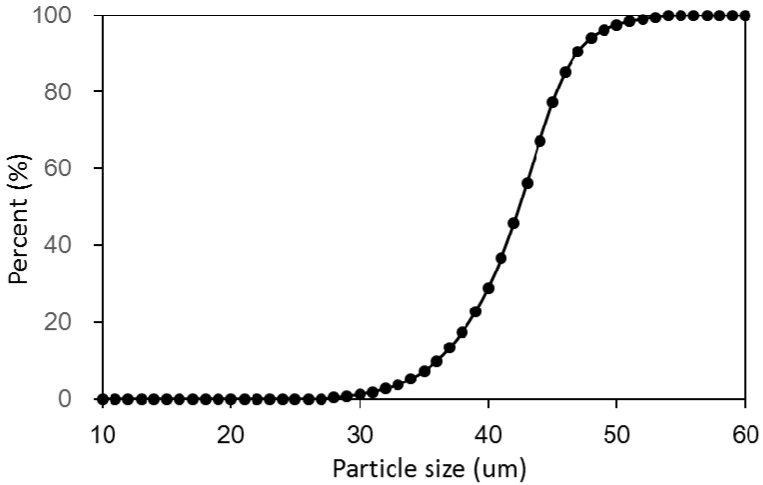
Particle size distribution of raw loess.

The sample examined in this study was deposited on Malan loess from the Quaternary age, which was taken from Lanzhou, China. This kind of loess is widely distributed in the Chinese Loess Plateau, of which it is a representative sample. The particle size curve of the loess is shown in Figure 1. The loess consisted of predominantly silt (about 91.3%) with a small amount of clay and sand (about 8.7%). The mean particle diameter was 0.034 mm and the coefficient of uniformity was 3.5. The loess had low plasticity. Some physical properties and chemical compositions are listed in Table 1.

**Figure 2:**
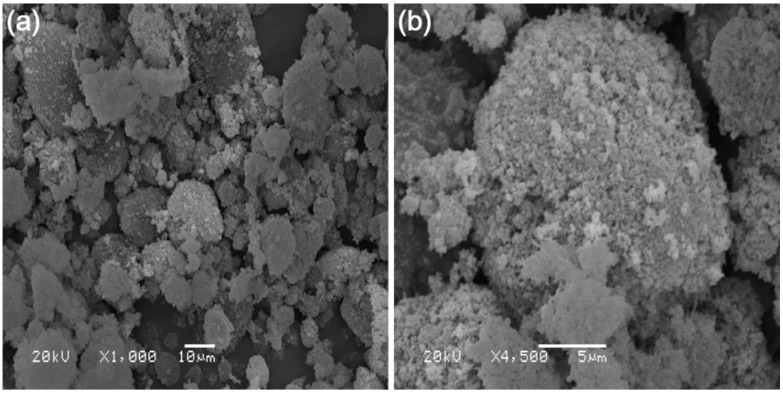
Scanning Electron Microscope SEM images of nano-SiO_2_ (**a**) amplification factor of 1000; (**b**) amplification factor of 4500.

The nano-SiO_2_ used in this study is commercially available, and came from the manufacturing industry (Changsha, China). Figure 2 shows the SEM images of nano-SiO_2_. As shown in Figure 2, the nano-SiO_2_ were isolated particles and agglomerated particles. The average size of the agglomerated particles was about 30 μm, although the average diameter of the isolated particle was 30 nm. The basic properties of nano-SiO_2_ are shown in Table 2.

### 2.2. Sample Preparation

The loess was first allowed to air dry at room temperatures of about 20 °C, which was passed through a sieve with a 0.5 mm aperture. This sieve was selected because the particle size of all soil was less than 0.5 mm. Distilled water was first added to the oven-dried, disaggregated loess to reach an initial water content of 15%, which was selected to obtain a uniform sample during compaction. Afterward, the samples were sealed and stored for 12 h at room temperature, which ensured a uniform distribution of moisture before packing for the following sample preparation.

The amount of dry nano-SiO_2_ selected was 0.2%, 0.4%, 0.8%, 1.0%, 1.5% and 2.0% of the total dry weight of the loess. The mixed samples were placed in a designed steel cylinder 50 mm in diameter and 100 mm in height. The cylindrical samples were prepared using a static compacted method with the help of a hydraulic jack and a steel holder. To achieve a uniform density, the samples were placed in five layers and each layer was compacted so that a designated dry density of 1.47 g/cm^3^ was achieved. The cylindrical samples were sealed using a plastic film and were cured for 7 days, 14 days, 28 days, and 60 days at room temperature.

### 2.3. Test Procedures

To obtain the mechanical behavior of the untreated and nano-SiO_2_ treated loess, the cylindrical samples after acquiring respective curing days were placed on an automatic loading machine (Lanzhou city, Gansu Province, China) with a maximum loading capacity of 100 kN and they were compressed at a constant rate of 0.1 mm/min. The smooth perspex plate was placed at the bottom of each specimen during all tests to minimize end effects. To examine the quality of the specimens and prevent possible errors, the unconfined compressive strength (UCS) of the specific samples was repeatedly tested two or three times. The average value of the repeated samples was used in data analysis. After compressive strength tests, the fractured samples were carefully collected for composition analysis and structure tests.

The macro-structures and micro-structures of the untreated and treated samples were examined. The macroscopic particle size distributions of all samples were determined using a Microtrac S3500 laser diffraction instrument (Lanzhou, China). The micro-morphology and micro-size were observed on the powder samples after metallization with gold powder using a JSM-5600LV scanning electron microscope (SEM) (Lanzhou, China). Meanwhile, nitrogen adsorption BET was conducted with an ASAP 2020 Plus physisorption analyzer (Lanzhou, China) on powder for the untreated and treated samples for their microscopic pore size. All BET tested samples were outgassed after 12 h before running analyses at a maximum temperature of 22 °C.

The mineralogical composition of the untreated and treated samples were examined by X-ray diffraction (XRD) for the specified samples. A Philips PW 3710 diffractometer (Lanzhou, China) was used for XRD analysis. The diffraction patterns were determined using Cu-Kα radiation with a Bragg angle (2θ) range of 5°–45°, running at a rate of 0.03°/s.

## 3. Results

### 3.1. Mechanical Behavior

**Figure 3:**
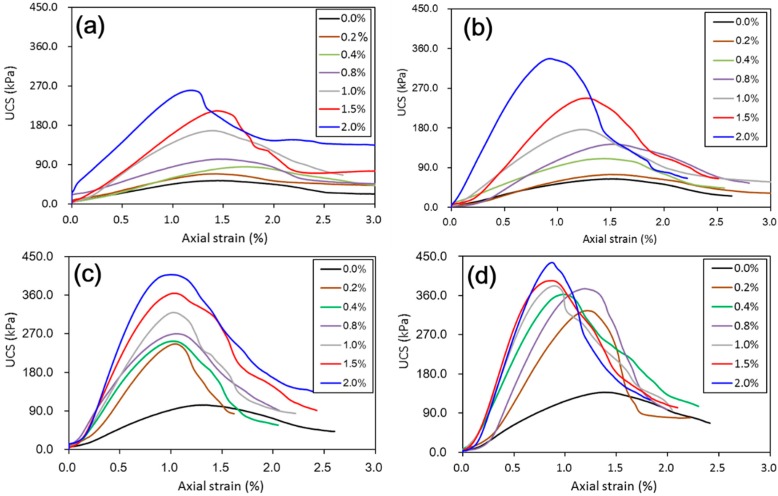
Effect of nano-SiO_2_ content and curing duration on stress–strain curves of treated loess. (**a**) 7 days; (**b**) 14 days; (**c**) 28 days; and (**d**) 60 days. (note: 0.0% denote untreated loess).

Figure 3 shows the stress-strain curves from unconfined compressive tests on untreated and treated loess with various amounts of nano-SiO_2_ content when subjected to different curing days. The test results are plotted here based on the axial strain measurement.

As shown in Figure 3, even at very low nano-SiO_2_ content, an increase in strength was observed for all treated samples with different curing days. However, the effect of nano-SiO_2_ content on the compressive behavior of the samples showed a clear difference in short-term (i.e., 7 days and 14 days) and long-term (i.e., 28 days and 60 days) curing duration in the presented study. For short-term curing duration, the ductile behavior was integrally maintained, with post-peak stress decreasing gradually with strain, which is a similar manner to that manifested by the untreated sample until nano-SiO_2_ content was greater than 2%; the peak stress increased observably after 1% of the nano-SiO_2_ content (see Figure 3a,b). With longer curing duration, i.e., 28 days and 60 days (see Figure 3c,d), all the treated samples became much more brittle than untreated samples. Meanwhile, they exhibited a quick drop after the peak stress with strain, which is more akin to the collapse failure of the meta-stable structure of saturated loess [22]. It is obvious that their peak stress increased significantly due to the addition of nano-SiO_2_; and the corresponding strain to peak stress decreased from 1.5% to approximately 1% (see Figure 3c,d).

**Figure 4:**
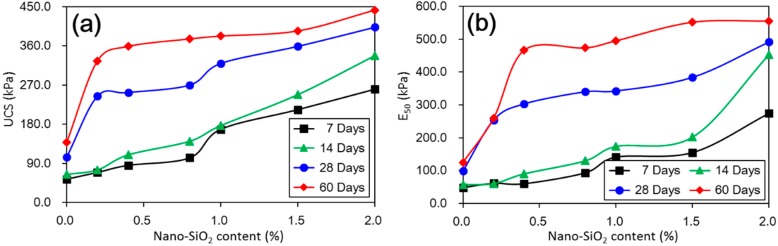
(**a**) Unconfined compressive strength (UCS) values of treated loess with different nano-SiO_2_ contents and curing days; (**b**) E_50_ values of treated loess with different nano-SiO_2_ contents and curing days.

Figure 4 shows the measured changes in UCS and E_50_ values with different nano-SiO_2_ contents and curing days. They presented a similar trend with increasing nano-SiO_2_ contents and curing days. The UCS and E_50_ values of nano-SiO_2_ treated loess were greater than that of untreated loess. The UCS and E_50_ values increased with higher nano-SiO_2_ content and with longer curing days. Moreover, the increase in the UCS and E_50_ values was more significant to a relatively longer curing day. Despite this, there are two different types of UCS and E_50_ values that changed, which is shown in Figure 4.

**Figure 5:**
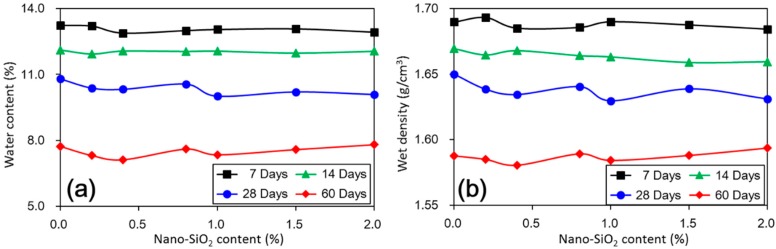
(**a**) Water content of treated loess with different nano-SiO_2_ contents and curing days; (**b**) Wet density of treated loess with different nano-SiO_2_ contents and curing days.

The first type was for tests with 7 days and 14 days of short-term curing. In this type, the UCS values of treated loess increased gradually with increasing nano-SiO_2_ content, which presented an almost linear trend. The second type was for tests with relatively longer curing duration, i.e., 28 days and 60 days. This presented an obviously nonlinear trend. The UCS values of the treated loess rapidly increased at very low (0.2%) nano-SiO_2_ content and after that only a slight increase with any further increase in nano-SiO_2_ content was observed. The changes could be attributed to a decrease in water content and wet density of nano-SiO_2_ treated loess, which is shown in Figure 5.

An unconfined compressive test is a common method for assessing the mechanical properties of stabilized soils [6]. Therefore, the UCS values can be used as an indicator to evaluate the efficiency of soil stabilization. According to criteria suggested by ASTM D4609-08 [23], an increase in UCS of 345 kPa or more due to treatment is considered effective. The test results indicated that the loess treated by 0.2% nano-SiO_2_ content after 60 days’ curing was almost effective. However, high nano-SiO_2_ contents treated loess did not achieve the ASTM criteria when the samples were cured for 7 and 14 days. This means that a sufficient curing day is more significant to the mechanical improvement of nano-SiO_2_ treated loess. This finding is consistent with those observed in lime-treated loess [6]. A similar finding was reported by Rogers, Glendinning [24]; who investigated the effect of lime addition to clay soils in the UK. These results all reveal that a small addition to seek full stabilization can be satisfied with enough curing duration for treated soils.

### 3.2. Macrotexture

**Figure 6:**
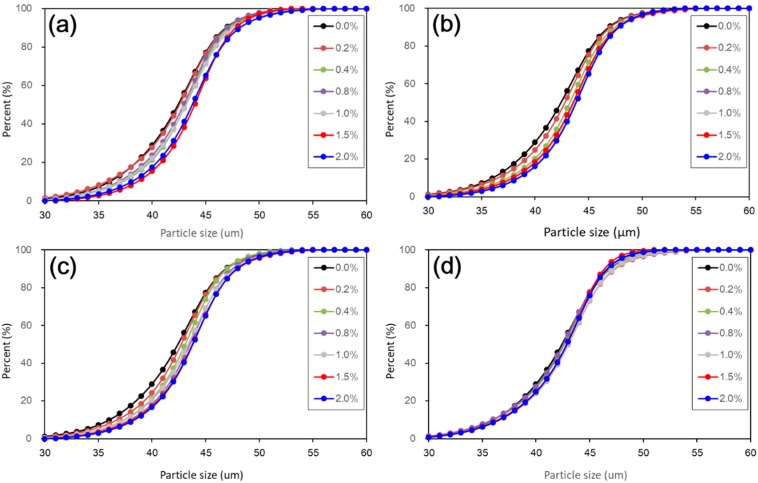
Effect of nano-SiO_2_ content and curing duration on cumulative particle size distribution of treated loess. (**a**) 7 days, (**b**) 14 days, (**c**) 28 days, and (**d**) 60 days.

Figure 6 shows the cumulative particle size distribution curves of untreated and treated loess with different nano-SiO_2_ contents and curing days. To facilitate a clearer view of particle size change, a linear abscissa was used, rather than a logarithmic abscissa. As a whole, the cumulative distribution curves show a continuous increase in particle size when nano-SiO_2_ contents increased. Nevertheless, during coarse particle development, there was still interesting and observed differences at different curing durations. At a given nano-SiO_2_ content, coarser particles formed when the curing duration was shorter. This means that the coarser particle development was the most striking. The degree of the coarser particle development slightly decreased only up to 28 days of curing. When curing duration reached 60 days, the particle size distribution curves of all the treated loess were much closer to that of untreated loess. This means that the coarse particle development became weak with longer curing duration.

**Figure 7:**
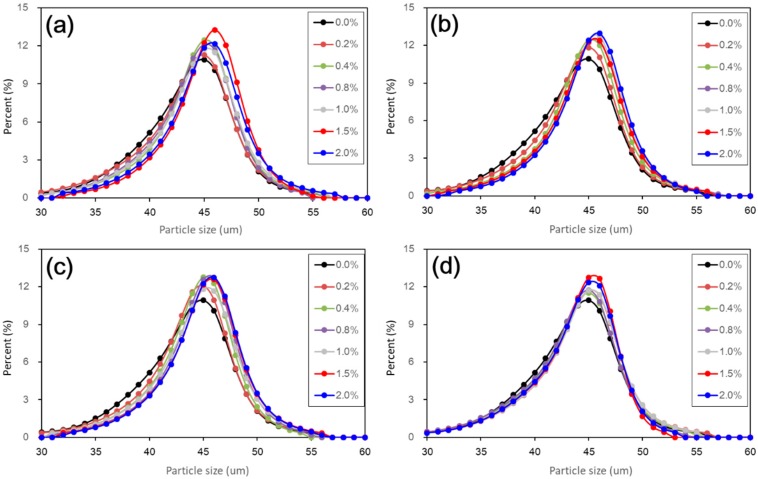
Effect of nano-SiO_2_ content and curing duration on frequent distribution curves of particle size of treated loess. (**a**) 7 days; (**b**) 14 days; (**c**) 28 days; and (**d**) 60 days.

Figure 7 shows the frequency distribution curves of particle size of untreated and treated loess with different nano-SiO_2_ contents and curing days. As shown in Figure 6, the results afford further details on the changes in particle size of nano-SiO_2_ treated loess. It can be seen that the 45 μm of particle size was a critical boundary. Before the boundary (i.e., 30~45 μm), the percent of finer particle sizes of the treated loess became gradually less with increasing nano-SiO_2_ content. When exceeding the boundary (i.e., 45~60 μm), the percent of coarser particle sizes of the treated loess became gradually greater with increasing nano-SiO_2_ content. Also, the peaks of particle size distribution of treated loess shifted slightly to the coarser side. These changes show that the nano-SiO_2_ treated loess resulted in coarser particle size compared to untreated loess and the modification was not very striking but it was observable, as shown in Figure 7.

Furthermore, the measurement of particle size by laser diffraction makes it easier to examine changes in the texture of treated soils. In addition, it can offer macroscopic evidence to better understand the microscopic characteristics [25,26], as it is not reliable to directly infer from micro-pore feature as suggested by Chew, Kamruzzaman [27]. The results from particle size distribution were aligned to the following SEM and BET microstructure observations.

### 3.3. Microstructure

**Figure 8:**
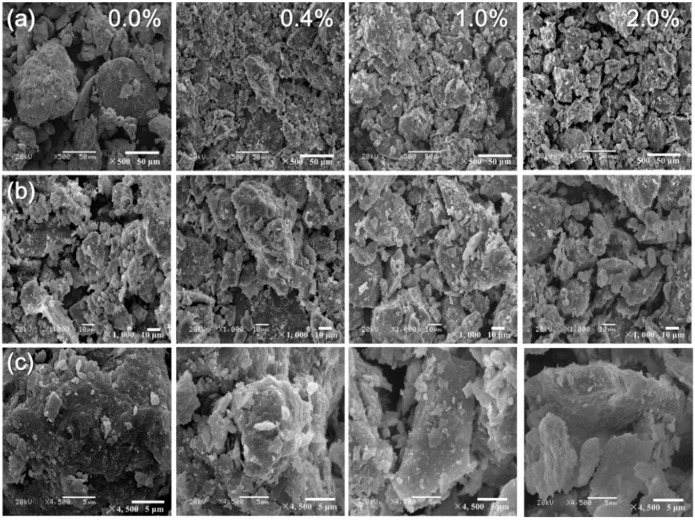
Microstructures of loess treated by 0.0%, 0.4%, 1.0%, and 2.0% nano-SiO_2_ contents after 7 days curing. (**a**) amplification factor of 500; (**b**) amplification factor of 1000; and (**c**) amplification factor of 4500.

Figure 8 shows the SEM images of the untreated loess with 0.4%, 1.0%, and 2.0% nano-SiO_2_ contents after seven days of curing. Figure 8a–c are at magnification factors of 500, 1000 and 4500, respectively. As shown in Figure 8a,b, two low magnification SEM images of untreated and treated loess revealed that the nano-SiO_2_ addition caused microstructural evolution with increasing content at the given curing duration. The low nano-SiO_2_ content addition first produced an observed filled effect in pores between particles or aggregations, which caused a decrease in porosity and an increase in the density degree of the treated loess. Afterward, the further increase in nano-SiO_2_ content promoted an obvious aggregated effect on the treated loess, which resulted in the formation of greater aggregations. When nano-SiO_2_ content was very high, the treated loess appeared in a state of looser packing and larger pores.

The interesting abnormity could be explained by high magnification SEM images (see Figure 8c). The SEM images recorded at high magnification show that nano-SiO_2_ addition formed a coating effect on the treated loess and the coating effect is more obvious to very high nano-SiO_2_ content treated loess. Essentially, the nano-SiO_2_ coating effect on treated loess played a dual role in structural changes. One direct role was that coating caused coarser particle size development, which was proven by the results from particle size distribution (as shown in Figure 6 and Figure 7). Another derivative effect was that coating restrains contact between particles or aggregations.

**Figure 9:**
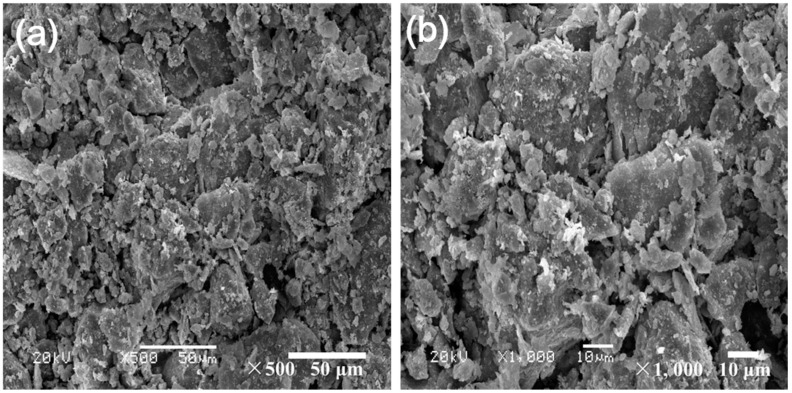
Microstructures of treated loess with 2.0% nano-SiO_2_ content after 28 days curing. (**a**) amplification factor of 500; (**b**) amplification factor of 1000.

Figure 9 shows the SEM images of the treated loess with 2.0% nano-SiO_2_ content after 28 days of curing. It can be seen that at a given nano-SiO_2_ content, a longer curing duration produces denser filling and smaller pores compared to a shorter one (Figure 8a,b). In addition, the nano-SiO_2_ particles itself formed agglomerations, which are easily observed in treated loess (Figure 8 and Figure 9).

**Figure 10:**
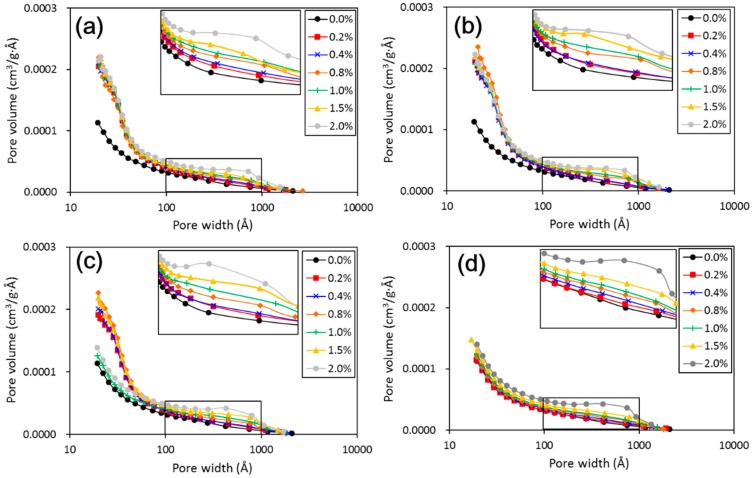
Pore volume versus pore width of treated loess at different nano-SiO_2_ content and curing duration. (**a**) 7 days; (**b**) 14 days; (**c**) 28 days; and (**d**) 60 days.

Figure 10 shows the pore size distribution curves of untreated and treated loess with different nano-SiO_2_ contents and curing days. It can be seen that the addition of nano-SiO_2_ resulted in a decrease in the amount of large pores. In all cases, there were two families of pore size, centered on about 50 Å of pore width and between 100 and 1000 Å of pore width. The two families belonged to inter-aggregated and intra-aggregated porosities, respectively. The relatively striking changes occurred mainly around 50 Å. Similarly, in the above-mentioned changes in macrostructure, after 28 days of curing the inter-aggregated porosity had almost disappeared in the nano-SiO_2_ treated loess (Figure 10d). The change of each pore family was slight, but its evolution was observable.

As a whole, the structure changes of treated loess observed from SEM images and BET analysis were matched with these changes in mechanical and textural properties. Their relations will be analyzed in the following discussion.

### 3.4. Mineralogical Composition

**Figure 11:**
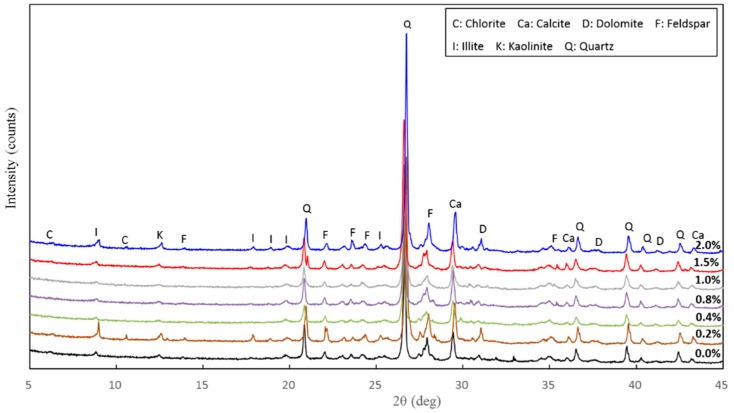
Mineralogical composition of the treated loess with different nano-SiO_2_ contents after curing for 7 days.

**Figure 12:**
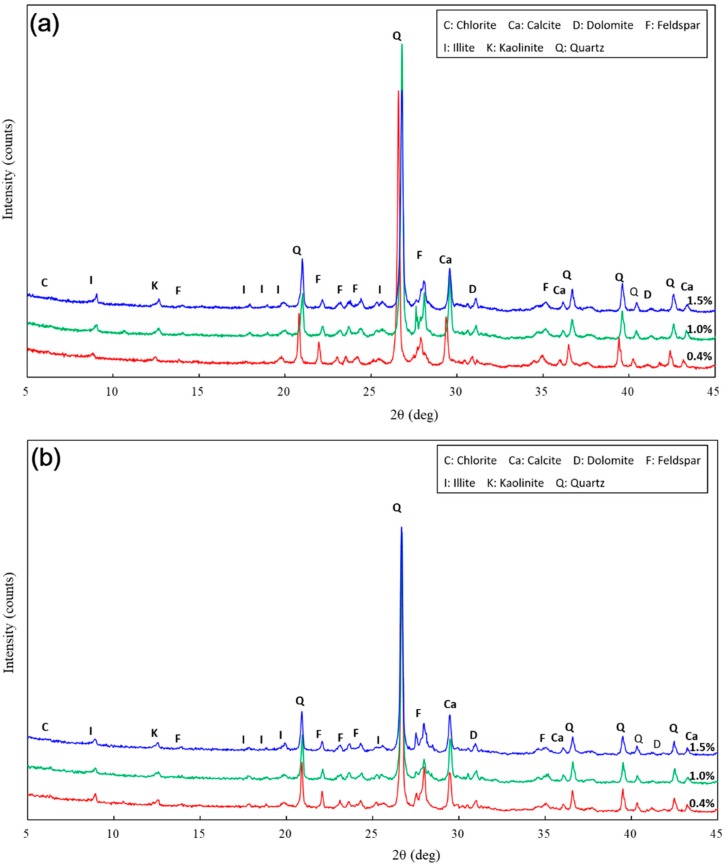
Mineralogical composition of the treated loess with specified nano-SiO_2_ contents after curing for 28 and 60 days.

Figure 11 shows the XRD diffractograms of loess with 0%, 0.2%, 0.4%, 0.8%, 1.0%, 1.5% and 2.0% nano-SiO_2_ content after 7 days of curing. Figure 12 shows the XRD diffractograms of treated loess for specified samples (i.e., 0.4%, 1.0% and 1.5% nano-SiO_2_ contents) after curing for 28 days and 60 days, respectively. It can be seen that the non-clay minerals identified consist mainly of quartz, calcite, and feldspar, while kaolinite, chlorite, and illite are the main clay mineral phases. Moreover, the effect of longer curing duration on mineralogical composition was insignificant to the treated loess at the same nano-SiO_2_ contents. The results support the observation of microstructures and macrostructures, which existed as only physical changes in particle size, fabric, and contact of the treated loess.

For all the examined samples, there were no observed changes in mineralogical compositions, while there was very little, if any, of the intensity and the full width at half maximum of the treated loess. The results revealed that a nano-SiO_2_ addition cannot cause the observed changes in mineralogical composition for treated samples, which may only produce a very slight alteration of the mineral structure. This means that physical alteration, such as filling, cementation, and coating occurred predominantly in the nano-SiO_2_ treated loess, which did give rise to a chemical reaction. This finding is consistent with those found in similar research performed by other authors [13,17].

## 4. Discussion

The above results have shown that the addition of nano-SiO_2_ can strikingly change the properties of treated loess. The compressive strength of nano-SiO_2_-treated loess increased with increasing nano-SiO_2_ contents at different curing days (see Figure 3). This resulted in an increase in USC and E_50_ values (see Figure 4). The decrease in water and volume supported the improvement of mechanical strength of nano-SiO_2_-treated loess (see Figure 5). The results from particle size distributions and SEM images show there was an obvious modification in structure caused by coarser particles, denser packing, and smaller pores (see Figure 6, Figure 7, Figure 8, Figure 9 and Figure 10). There was a similar trend in particle size and pore size after 28 days of curing. This means that the development of coarse particles and denser packing became weak with increasing curing duration. The particle size distributions and SEM images afforded a nice observation of macrostructure and microstructure, respectively. However, the BET analyses permitted better observation of the pore families and their evolution over curing duration. Meanwhile, the mineralogical components have proven that the changes in mechanical and structural properties of nano-SiO_2_-treated loess are physical alterations rather than chemical reactions (see Figure 11 and Figure 12). The physical densification induced a strengthening effect on loess, which has also been observed its shear strength [28].

There was a close relationship between macroscopic behaviors (such as, mechanical strength, density and water content) and microscopic characteristics (such as, structure and mineralogy) of treated soils [25,26,27]. The SEM images results (see Figure 8 and Figure 9) confirmed that the addition of a nano-SiO_2_ particle caused the filling of porous areas and the formation of greater aggregations and consequently caused coarser particle size and more interparticle contact. To some degree, the nano-SiO_2_ particle itself was also beneficial to the aggregation formation [13]. This agglomerated effect can be attributed to large surface areas and high surface reactivity of the nano-SiO_2_ particle. As a result, the nano-SiO_2_-treated loess produced stronger mechanical strength. Moreover, previous studies have found that loess with aggregates has a relatively higher strength [2,22].

There are two types of change trends in mechanical strength of treated loess. The different styles and trends are dependent on the reactive activity of nano-SiO_2_ at different curing durations. Under short-term curing duration, the ductile behavior and linear increase in strength are related to the evolution of the structure due to nano-SiO_2_ addition. As observed in Figure 8, the nano-SiO_2_ filling effect occurred first in pores between particles or aggregations and then came an obvious coating effect to single particle due to an ongoing addition. Nevertheless, the reactive activity of nano-SiO_2_ was relatively weak to treated loess in the whole process. However, the structural changes were not a transient activity in processing. Under long-term curing duration, the brittle behavior and nonlinear increase in strength were attributed to stronger structure effect, which caused denser packing and smaller pores (Figure 5, Figure 6, Figure 7, Figure 8, Figure 9 and Figure 10). We inferred that this is related to the nano-SiO_2_ self-properties, due to large surface areas and high surface reactivity. The differences further show that various additives are time-dependent in improving the performance of treated soils. Therefore, for effective cost control in particle engineering, it is crucial that there is enough curing time to guarantee treated soils an appropriate addition content.

## 5. Conclusions

The present paper examined the changes in mechanical, mineralogical, and structural properties of nano-SiO_2_ treated loess with different contents and curing days. The compressive test results showed that mechanical strength of nano-SiO_2_-treated loess gradually improves by increasing content and curing days. The accumulative increase in mechanical strength can be attributed to the coarser particle, denser packing and smaller pore processes in nano-SiO_2_-treated loess. These changes in microscopic characteristics can be proven by the evidence from particle size, SEM, and BET. Furthermore, the changes in dry density and water content, along with the unchanged mineralogy, support the idea that the increase in mechanical strength of treated loess resulted from their physical structure modification rather than chemical alteration due to the addition of nano-SiO_2_. The results presented in this research have shown that nano-SiO_2_ may serve as a cost-effective additive in loess stabilization. Meanwhile, a small addition to seek full stabilization can be satisfied for nano-SiO_2_ treated loess when curing duration is long enough. Additionally, there was a close relationship between microscopic characteristics (mineralogy, microstructure and microspore) and macroscopic behaviors (strength, texture, state, etc.). The macroscopic behaviors of loess significantly depend on its microscopic characteristics. Thus, the linked relations can facilitate the understanding of the effect of nano-SiO_2_ on loess properties and their complicated interactions.

In addition, there is a strong need to conduct systematic laboratory experiments and particular field research into various nanomaterials that may be used as stabilizing additives to facilitate a better understanding for practical applications. Laboratory measurements are important for providing useful post hoc estimates for practical applications but field evaluations of the nano-materials are still not enough. Hence, there is a need to conduct an in-situ evaluation and a longer curing duration for nano-SiO_2_-treated loess.

## Figures and Tables

**Table 1:** Physical properties and chemical compositions of loess used in this study.

*Physical Properties*	Value
Specific gravity (Gs)	2.71
Liquid limit (%)	27.98
Plastic limit (%)	17.45
Plasticity index (%)	10.53
Specific surface area (m^2^/g)	27.5
Cation exchange capacity (meq/100 g)	3.5
***Chemical compositions (weight%)***	
P_2_O_5_	0.16
TiO_2_	0.65
SiO_2_	54.73
Al_2_O_3_	11.81
Fe_2_O_3_	4.57
MnO	0.066
MgO	2.82
CaO	9.61
Na_2_O	2.18
K_2_O	2.47
LOI	10.78
Total	99.84

**Table 2:** Basic properties of nano-SiO_2_ used in this study.

Property	Value
Average diameter (nm)	30
Purity (%)	≥99.8
Density (g/cm^3^)	<0.15
Specific surface area (m^2^/g)	300
Color	White
Morphology	Spherical solid
